# A de novo* TOP2B* variant associated with global developmental delay and autism spectrum disorder

**DOI:** 10.1002/mgg3.1145

**Published:** 2020-01-17

**Authors:** Takuya Hiraide, Seiji Watanabe, Tomoko Matsubayashi, Kumiko Yanagi, Mitsuko Nakashima, Tsutomu Ogata, Hirotomo Saitsu

**Affiliations:** ^1^ Department of Biochemistry Hamamatsu University School of Medicine Hamamatsu Japan; ^2^ Department of Pediatrics Hamamatsu University School of Medicine Hamamatsu Japan; ^3^ Department of Pediatrics Izu Medical and Welfare Center Izunokuni Japan; ^4^ Department of Pediatric Neurology Shizuoka Children's Hospital Shizuoka Japan; ^5^ Department of Genome Medicine National Center for Child Health and Development Tokyo Japan

**Keywords:** autism spectrum disorder, global developmental delay, *TOP2B*, whole‐exome sequencing

## Abstract

**Background:**

*TOP2B* encodes type II topoisomerase beta, which controls topological changes during DNA transcription. *TOP2B* is expressed in the developing nervous system and is involved in brain development and neural differentiation. Recently, a de novo missense *TOP2B* variant (c.187C>T) has been identified in an individual with neurodevelopmental disorder (NDD). However, the association between *TOP2B* variants and NDDs remains uncertain.

**Methods:**

Trio‐based whole‐exome sequencing was performed on a 7‐year‐old girl, presenting muscle hypotonia, stereotypic hand movements, epilepsy, global developmental delay, and autism spectrum disorder. Brain magnetic resonance images were normal. She was unable to walk independently and spoke no meaningful words.

**Results:**

We found a de novo variant in *TOP2B* (NM_001330700.1:c.187C>T, p.(His63Tyr)), which is identical to the previous case. The clinical features of the two individuals with the c.187C>T variant overlapped.

**Conclusion:**

Our study supports the finding that *TOP2B* variants may cause NDDs.

## Graphical abstract

Here we describe a Japanese girl with a de novo TOP2B variant c.187C>T, p.(His63Tyr) detected by trio‐based exome sequencing. She is the second case with a recurrent c.187C>T, p.(His63Tyr) TOP2B variant and shared similar neurodevelopmental phenotypes with the previous case.

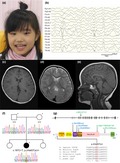

## 1 INTRODUCTION

Neurodevelopmental disorders (NDDs) are genetically heterogeneous and recent whole‐exome sequencing (WES) studies have revealed the importance of de novo variants in NDDs (Ku et al., 2013). Variants in several genes involved in transcription cause global developmental delay (GDD) and autism spectrum disorder (ASD) (De Rubeis et al., 2014). These variants are likely to affect relevant gene expressions, which impairs various functional pathways in neural development (De Rubeis et al., 2014).

DNA Topoisomerase II Beta (*TOP2B*) is a DNA topoisomerase that controls and alters the topologic states of DNA during transcription (Austin et al., 2018). *TOP2B* is expressed in human fetal brain and is present in both proliferative and post mitotic cells (Harkin et al., 2016). Knock‐out mice studies indicated that *Top2b* play important roles both in brain development and neural differentiation (Edmond, Hanley, & Philippidou, 2017; Lyu & Wang, 2003). Recently a de novo* TOP2B* variant (c.187C > T, p.(His63Tyr)) was found in a patient with GDD (Lam, Yeung, & Law, 2017); however, the association between *TOP2B* variants and NDDs remains uncertain. Here, we report an individual with GDD and ASD with a de novo* TOP2B* variant. Our case may support a relationship between *TOP2B* and NDDs.

## 2 CLINICAL REPORT

**Figure 1:**
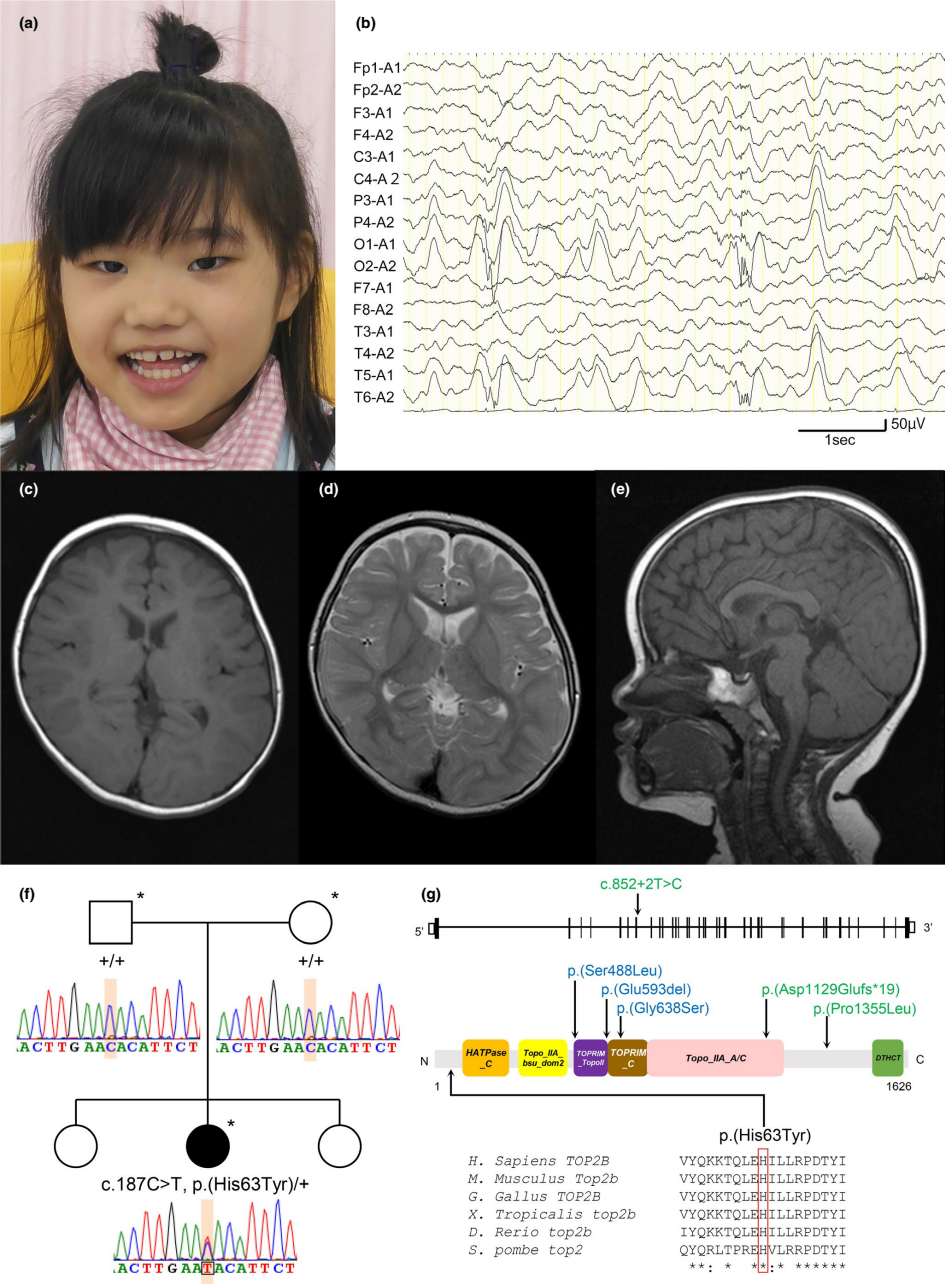
(a) Clinical photograph taken at 7 years and 3 months old. (b) Interictal electroencephalogram at 6 years and 7 months old. Electroencephalogram showed spike‐and‐wave discharges in the right occipital area. (c–e) Brain MRI taken at 1 year 9 months old. (c) Axial image in T1‐weighted image, (d) axial image in T2‐weighted image and (e) sagittal image in T1‐weighted image reveal no delayed myelination or abnormality. (f) Familial pedigrees and Sanger sequencing of *TOP2B*. Asterisks denote members who underwent whole exome sequencing. +, wild‐type allele. (g) Schematic presentation of the *TOP2B* gene (upper) and protein structure (lower). The TOP2B protein comprises six domains: Histidine kinase/HSP90‐like ATPase (*HATPase_C*), DNA topoisomerase, type IIA, subunit B, domain 2 (*Topo_IIA_bsu_dom2*), DNA topoisomerase 2, TOPRIM domain (*TOPRIM_TopoII*), C‐terminal associated domain of TOPRIM (*TOPRIM_C*), DNA topoisomerase, type IIA, subunit A/C‐terminal (*Topo_IIA_A/C*) and DTHCT (from InterPro). Previously reported TOP2B variants are depicted above (previously reported in immunodeficiency (Broderick et al., 2019), blue; previously large cohort reported in NDDs (Kosmicki et al., 2017), green). The TOP2B variants in our case are shown below. Multiple amino acid sequences of TOP2B were aligned using the ClustalW tool

After 39 weeks and 6 days of gestation without asphyxia, a Japanese girl was born to nonconsanguineous healthy parents as their second child. There was no family history of NDDs. Her birth weight, body length, and head circumference were 3,054 g (0.01 standard deviation [*SD*]), 51 cm (1.0 *SD*), and 33 cm (−0.24 *SD*), respectively. She had no dysmorphic features (Figure 1a). Her developmental milestones were delayed: head control at 6 months old, smiling at 10 months old, sitting independently at 1 year and 2 months old and crawling at 1 year and 8 months old. Muscle hypotonia was observed since infancy. Stereotypic hand movements such as handwashing and putting hands in her mouth have been observed since she was 2 years old. Poor eye contact and no interest in toys were noticed. She was diagnosed with ASD in early childhood. Hearing loss was not recognized. At 6 years and 3 months old, tonic‐clonic seizures developed and antiepileptic drugs were initiated. Electroencephalogram revealed epileptiform discharges in the right occipital area (Figure 1b). Brain magnetic resonance imaging (MRI) at 1 year 9 months old showed normal brain structure (Figure 1c‐e). The final physical examination at 7 years 3 months showed a body weight of 20 kg (−0.9 *SD*), height 114 cm (−1.4 *SD*), and head circumference 49.5 cm (−1.4 *SD*). She was unable to speak any meaningful words. She could walk with support but was unable to stand without assistance. Her developmental age was assessed as 10 months old. Stereotypic hand movements decreased. She could grab things but it was difficult to pinch. Muscle hypotonia was seen, predominantly in the trunk.

## 3 GENETIC ANALYSIS

This study was approved by the Institutional Review Board Committee at Hamamatsu University School of Medicine. After receiving written informed consent, genomic DNA extracted from blood leukocytes from the patient and her parents were analyzed using WES. Data processing, variant calling, annotation, and filtering were performed as described previously (Hiraide et al., 2019). Using trio‐based WES data, we identified a candidate de novo variant in *TOP2B* (NM_001330700.1:c.187C>T, p.(His63Tyr)), which was validated by Sanger sequencing (Figure 1f). This variant was absent in our 218 in‐house Japanese control exome data and public databases, including the Genome Aggregation Database (gnomAD, see http://gnomad.broadinstitute.org/; accessed November 2019) and the Integrative Japanese Genome Variation Database (4.7KJPN, see https://ijgvd.megabank.tohoku.ac.jp/). This variant was predicted to be deleterious using in silico pathogenicity prediction tools (Table S1). We also identified three other de novo variants (*THEMIS2*, *OR4C3* and *SRRM2*) and two compound heterozygous variants (*LRRFIP1*; Tables S1 and S2), but both two variants in *LRRFIP1* were predicted to be benign. Two genes (*THEMIS2* and *OR4C3*) show low pLI score, which suggests tolerance to loss of function variants. No obvious association with human diseases has been reported in these two genes. Missense variants in *SRRM2* are associated with NDDs and ASD (Iossifov et al., 2014; Jin et al., 2017; Takata et al., 2018). However, the pathogenicity of missense variants in *SRRM2* is uncertain because the Z score of *SRRM2* is negative (=−6.28) in gnomAD database, which suggests tolerance to missense variations. Although we examined copy number variants using the eXome‐Hidden Markov Model (XHMM) and the methods developed by Nord et al. (Fromer et al., 2012; Nord, Lee, King, & Walsh, 2011), no candidate CNVs were detected. These findings suggested that this *TOP2B* variant was the most possible causative variant in this case.

## 4 DISCUSSION

We identified a de novo* TOP2B* variant associated with NDD. Large cohort investigations for congenital heart disease with NDDs or developmental disorders reported three de novo* TOP2B* variants, but their clinical descriptions are not available (Figure 1g) (Kosmicki et al., 2017). Lam et al. described an individual with a de novo c.187C>T *TOP2B* variant (Lam et al., 2017); this is the second case with the c.187C>T *TOP2B* variant. Both patients showed similar neurodevelopmental phenotypes, such as truncal hypotonia, GDD, and ASD (Table 1). Craniofacial, ocular, and skeletal abnormalities were only observed in the previous case. Recently, three heterozygous *TOP2B* variants were found in three unrelated families suffering from syndromic B‐cell immunodeficiency with facial dysmorphism, genital malformations, and limb anomalies (Figure 1g) (Broderick et al., 2019). Two of three families with immunodeficiency syndromes showed developmental delay and growth impairment, but details of the neurological symptoms were not available (Broderick et al., 2019). However, episodes of recurrent infections were not noted in both individuals with the c.187C>T variant. These findings suggested that de novo* TOP2B* variants are strongly associated not only with NDDs but also various congenital anomalies.

**Table 1:** Clinical findings of individuals with *TOP2B* variants (Lam et al., 2017)

Individuals	This study	Lam et al.
Variant	c.187C > T	c.187C > T
	p.(His63Tyr)	p.(His63Tyr)
Status	de novo	de novo
*Sex, age*	F, 7 years	F, 15 years
Dysmorphic features	−	−
Hypotonia	+	+
Scoliosis	−	+
Stereotypic hand movements	+	−
Microcephaly	−	+
Growth delay	−	+
Global developmental delay	+	+
Motor development	Walk with support	Walk with support
Intellectual disability	Severe	Severe
Speech development	No word	No word
Autistic behavior	+	+
Seizures	+	−
Abnormal electroencephalogram	+	N.A.
Abnormal brain MRI	−	−
Ophthalmological disorder	−	Strabismus, myopia and astigmatism

Abbreviations: MRI, magnetic resonance imaging; N.A., not assessed or not available.

The TOP2B enzyme binds to gene regulatory regions and alters the topologic states of DNA in transcription and replication. TOP2B regulates gene transcription and generates transient DNA double‐strand breaks (Ju et al., 2006). Interestingly, all three *TOP2B* variants found in B‐cell immunodeficient cases were located at the topoisomerase‐primase (TOPRIM) domain, which controls DNA cleavage by binding catalytic metal ions (Figure 1g) (Bax, Murshudov, Maxwell, & Germe, 2019). Broderick et al. indicated that variants in the TOPRIM domain of Top2b reduced the expression of some genes that encoded the B cell‐specific transcription factors with a dominant negative effect that affected multiple stages of B‐cell development (Broderick et al., 2019). However, the variants associated with NDDs spread throughout the gene (Figure 1g). In a mice study, the absence of *Top2b* induced poor cerebral cortex development and perinatal death (Lyu et al., 2003). Moreover, TOP2B silencing in human mesenchymal stem cells affected the expression pattern of many genes associated with neuronal differentiation (Isik et al., 2015). These findings implied that the dysfunction of TOP2B may induce dysregulation of the gene expression involved in neural development and differentiation and lead to neuropsychiatry disorders.

In conclusion, we identified a case with a recurrent de novo* TOP2B* variant. Although our case supports the pathogenicity of de novo* TOP2B* variants in NDDs, further investigations will be necessary to establish the causal relationship between de novo* TOP2B* variants and NDDs.
