# Supplementary material for: A de novo TOP2B variant associated with global developmental delay and autism spectrum disorder
Source: Mol Genet Genomic Med. 2020 Jan 17;8(3):e1145. doi: 10.1002/mgg3.1145 (PMC7057084; doi:10.1002/mgg3.1145)
Supplement: Supplementary file 1 [file MGG3-8-e1145-s001.docx]

**Table S1. Candidate variants identified by Trio-WES**

| Chr | Gene | Variant | Origin | gnomAD | pLI | SIFT | PP2  HVAR | CADD phred | M-CAP | GERP | Mutation  Taster |
| --- | --- | --- | --- | --- | --- | --- | --- | --- | --- | --- | --- |
| 3 | *TOP2B* | NM_001330700.1:c.187C>T,  p.(His63Tyr) | *de novo* | − | 1.000 | 0 | 1.000 | 27.4 | 0.018 | 5.54 | 1 |
| 1 | *THEMIS2* | NM_001105556.3:c.733_734delinsCC,  p.(Lys245Pro) | *de novo* | − | 0.002 | 0.003 | 0.881 | N.A. | N.A. | N.A. | N.A. |
| 11 | *OR4C3* | NM_001004702.1:c.191_195delinsGGGGG,  p.(Val64_Cys65delinsGlyGly) | *de novo* | − | 0 | N.A. | N.A. | N.A. | N.A. | N.A. | N.A. |
| 16 | *SRRM2* | NM_016333.4:c.7982A>C,  p.(Lys2661Thr) | *de novo* | − | 1.000 | N.A. | 0.981 | 26.9 | 0.009 | 5.19 | 0.917 |
| 2 | *LRRFIP1* | NM_001137552.1:c.1651G>A,  p.(Asp551Asn) | Compound heterozygous | 0.00001594 | 0.020 | 0.891 | 0.001 | 1.672 | 0.004 | −2.21 | 1 |
| 2 | *LRRFIP1* | NM_001137552.1:c.2050G>A,  p.(Val684Ile) | Compound heterozygous | 0.0001203 | 0.020 | 0.328 | 0.001 | 0.021 | 0.003 | −5.3 | 1 |

gnomAD (the Genome Aggregation Database, <http://gnomad.broadinstitute.org/>), SIFT (Sorting Intolerant From Tolerant): <http://sift.jcvi.org/>, Polyphen-2 Hum Var: <http://genetics.bwh.harvard.edu/pph2/>, CADD (Combined Annotation–Dependent Depletion): <http://cadd.gs.washington.edu/score>, M-CAP (Mendelian Clinically Applicable Pathogenicity): <http://bejerano.stanford.edu/mcap/index.html>, GERP (Genomic Evolutionary Rate Profiling): <http://mendel.stanford.edu/SidowLab/downloads/gerp/>, MutationTaster: <http://www.mutationtaster.org/>. N.A., not available.

**Table S2. Variants classification following ACMG guideline**

| **Gene** | **Variant** | **Evidence of pathogenicity** | | | | **classification** |
| --- | --- | --- | --- | --- | --- | --- |
|  |  | **Very strong** | **Strong** | **Moderate** | **Supporting** |  |
| *TOP2B* | c.187C>T, p.(His63Tyr) | Not applicable | PS2 | PM2 | PP3, PP5 | Likely pathogenic |
| *THEMIS2* | c.733_734delinsCC, p.(Lys245Pro) | Not applicable | PS2 | PM2 | Not applicable | Likely pathogenic |
| *OR4C3* | c.191_195delinsGGGGG,  p.(Val64_Cys65delinsGlyGly) | Not applicable | PS2 | PM2 | Not applicable | Likely pathogenic |
| *SRRM2* | c.7982A>C, p.(Lys2661Thr) | Not applicable | PS2 | PM2 | Not applicable | Likely pathogenic |
| *LRRFIP1* | c.1651G>A, p.(Asp551Asn)/ c.2050G>A, p.(Val684Ile) | Not applicable | Not applicable | Not applicable | Not applicable | Uncertain Significance |

PS2: *De novo* (both maternity and paternity confirmed) in a patient with the disease and no family history.

PM2: Absent from controls (or at extremely low frequency if recessive) in Exome Sequencing Project, 1000 Genomes Project, or Exome Aggregation Consortium.

PP3: Multiple lines of computational evidence support a deleterious effect on the gene or gene product (conservation, evolutionary, splicing impact, etc.)

PP5: Reputable source recently reports variant as pathogenic, but the evidence is not available to the laboratory to perform an independent evaluation.
